# Periodontitis associates with species-specific gene expression of the oral microbiota

**DOI:** 10.1038/s41522-021-00247-y

**Published:** 2021-09-23

**Authors:** Daniel Belstrøm, Florentin Constancias, Daniela I. Drautz-Moses, Stephan C. Schuster, Mark Veleba, Frédéric Mahé, Michael Givskov

**Affiliations:** 1 Section for Periodontology and Oral Microbiology, Department of Odontology, Faculty of Health and Medical Sciences, University of Copenhagen, Copenhagen, Denmark; 2 Singapore Centre for Environmental Life Sciences Engineering, Nanyang Technological University, Singapore, Singapore; 3 BGPI, Univ. Montpellier, CIRAD, INRA, Montpellier SupAgro, Montpellier, France; 4 Costerton Biofilm Center, Department of Immunology and Microbiology, Faculty of Health and Medical Sciences, University of Copenhagen, Copenhagen, Denmark; 5 Present Address: Laboratory of Food Biotechnology, Department of Health Sciences and Technology, ETH Zürich, Zürich, Switzerland

**Keywords:** Microbiome, Biofilms

## Abstract

The purpose of the present investigation was to characterize species-specific bacterial activity of the oral microbiota in periodontitis. We tested the hypotheses that chronic inflammation, i.e., periodontitis, associates with bacterial gene expression of the oral microbiota. Oral microbial samples were collected from three oral sites—subgingival plaque, tongue, and saliva from patients with periodontitis and healthy controls. Paired metagenomics and metatranscriptomics were used to perform concomitant characterization of taxonomic composition and to determine species-specific bacterial activity as expressed by the ratio of specific messenger RNA reads to their corresponding genomic DNA reads. Here, we show the association of periodontitis with bacterial gene expression of the oral microbiota. While oral site was the main determinant of taxonomic composition as well as bacterial gene expression, periodontitis was significantly associated with a reduction of carbohydrate metabolism of the oral microbiota at three oral sites (subgingival plaque, tongue, and saliva). Data from the present study revealed the association of periodontitis with bacterial gene expression of the oral microbiota. Conditions of periodontitis was associated with bacterial activity of local subgingival plaque, but also on tongue and the salivary microbiota. Collectively, data suggest that periodontitis associates with impaired carbohydrate metabolism of the oral microbiota. Future longitudinal and interventional studies are warranted to evaluate the potential pathogenic role of impaired bacterial carbohydrate metabolism not only in periodontitis but also in other diseases with low-grade inflammation, such as type 2 diabetes mellitus.

## Introduction

The oral cavity harbors the second most diverse microbiota found in the human organism^1^, and the open-ended ecosystem of the oral cavity entails that the oral microbiota is constantly stressed by internal and external perturbations^2^. The prime constant ecological determinant is O_2_ availability, which is critically different across oral sites, with the buccal mucosa and the tongue surface as examples of areas with extreme anaerobic and aerobic conditions, respectively^3^. As a consequence of microbial adaptation to the ecological conditions being present at different oral sites, considerable taxonomic variations are observed at various oral sites^4,5^. As the taxonomic composition of the oral microbiota has been characterized in depth in oral health, the oral cavity is an ideal model system to study bacterial activity of polymicrobial biofilms thriving in areas with different ecological conditions.

Periodontitis, which is a chronic inflammatory disease of the tooth supporting tissue, is an example of a constant ecological determinant, which affect not only the composition of the local subgingival microbiota but also the salivary microbiota^6,7^. Likewise, cigarette smoking and electronic cigarettes are transient external stressors on the oral microbiota, all with known effect on bacterial gene expression in subgingival plaque^8,9^. We have recently used a contemporary paired metagenomic and metatranscriptomic approach to reveal that periodontitis is associated with characteristics of salivary bacterial activity different from that of oral health^10^. However, as we did only characterize bacterial activity of the salivary microbiota, we were not able to evaluate if the salivary characteristics identified were associated with the same characteristics of local oral biofilms such as subgingival plaque and tongue.

In the present investigation, we therefore collected multiple samples—subgingival plaque, tongue scrapings and saliva—which are characterized by substantially different ecological conditions and microbial communities^11–13^. Metagenomics and metatranscriptomics were employed to perform concomitant characterization of taxonomic composition as well as specific bacterial gene expression profiles. Furthermore, pairing of metagenomic and metatranscriptomic data enabled us to determine species-specific bacterial activity as expressed by the ratio of messenger RNA to the corresponding genomic DNA. In other words, the resolution of our paired deep sequencing approaches enable us to discriminate between members of the microbiota, with overexpression of important pathways (log10(RNA/DNA) > 0), as compared to members with less expression (log10(RNA/DNA) < 0).

We tested the hypothesis that presence of chronic inflammation, i.e., periodontitis, associates with bacterial gene expression not only locally (subgingival plaque) but also at other sites of the oral cavity (tongue and saliva).

## Results

### Sequence processing

A total of seven samples failed quality controls, which means that 59 samples were included in downstream DNA and RNA analyses. From a total of 59 microbial samples (19 subgingival plaque samples, 18 tongue biofilm, 22 saliva samples), 1215 M DNA sequences (per-sample mean 20.59 M; range 15.14–35.05 M) and 3093 RNA sequences (per-sample mean 52.43 M; range 39.97–74.65 M) were generated.

In total, 612, 851 M (mean 10.38 M; range 0.52–21.67 M) DNA read pairs and 95, 943 M (mean 1.62 M; range 0.27–2.9 M) RNA read pairs were used after quality filtering, rRNA removal, and host (i.e., human) contamination for further analysis. A comparable average number of DNA and RNA sequences passing quality control were identified in samples from each site: subgingival plaque (DNA: 7.86 M, RNA: 1.16 M), tongue (DNA: 16.84 M, RNA: 1.62 M), and saliva (DNA: 7.29 M, RNA: 2.03 M), and as well as in patients with periodontitis (DNA: 9.94 M, RNA: 1.49 M) versus orally healthy controls (DNA: 10.84 M, RNA: 1.76 M).

### Microbial diversity and taxonomic composition is influenced by site and periodontitis

**Fig. 1:**
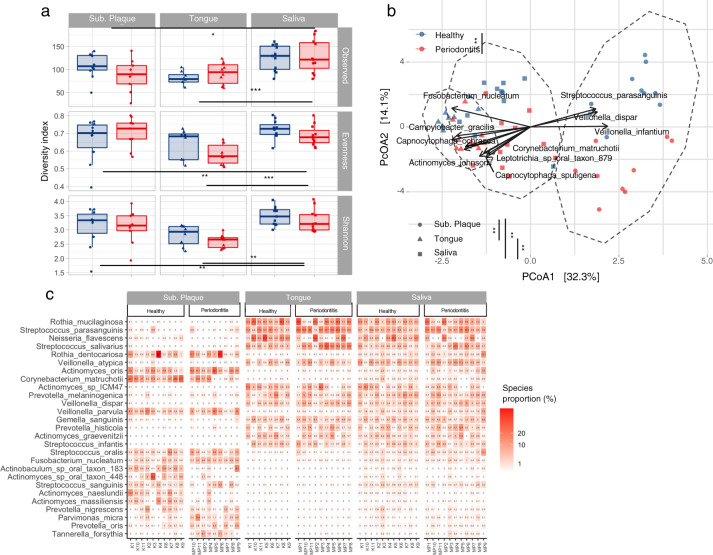
Diversity and taxonomic composition of oral microbiota. **a** α-diversity patterns as measured by observed richness, Pielou’s evenness and Shannon diversity index. **b** β-diversity as summarized by Aitchison distance and visualized using PCoA ordination. **c** Top ten species identified in oral site x health status combinations (27 species in total). Color indicates heath status (blue: healthy controls versus red: periodontitis group), and shape reflects oral site. Significant differences were assessed using Kruskal–Wallis and PERMANOVAS tests for α and β-diversity, respectively. Significant FDR-adjusted *p* values were indicated as follows: *0.05 > *p* > 0.01 **0.01 > *p* > 0.001 ***p < 0.001. Boxplots display first quartile, median, and third quartile and whiskers represent 1.5 times the interquartile range from the first and third quartiles. The top 12 species significantly (*p* < 0.05) correlated among the PCoA 1 and 2 axes were displayed using vegan::envfit() function. Microbial taxonomic profiles are based on MetaPhlAn v3.0.1—default parameters.

A comparison of α-diversity as measured by Shannon index, species richness, and evenness revealed significant differences based on site, with higher α-diversity in subgingival plaque and saliva, as compared to tongue biofilm. However, α-diversity at each site was not influenced by health status (Fig. 1a). Also, species community structure quantified by Atchinson distance was more influenced by site (PERMANOVA, *R*^2^ = 0.33, *p* = 0.001) than health status (PERMANOVA, *R*^2^ = 0.07, *p* = 0.001, Fig. 1b) and the interaction term between oral site and health status was not significant indicating that health status is consistent with species community structure. Figure 1c displays relative abundance of top 27 predominant bacterial species across the three sites and their health status. The microbiota of each site was characterized with a specific species combinations being clearly different from the other sites. On the other hand, subgingival plaque was the only site, where the predominant species were influenced by periodontitis, as exemplified by *Tannerella forsythia*, which was present in almost all samples from patients with periodontitis, and in only one sample from the healthy controls. A comparison of relative abundance of all species across all samples revealed 25 species, with significantly different relative abundances in samples from patients with periodontitis versus healthy controls, with higher relative abundances of periodontal pathogens such as *Filifactor alocis, Parvimonas micra, Prevotella intermedia*, *Treponema denticola*, and *T. forsythia* identified in samples from patients with periodontitis, and higher relative abundances of *Actinomyces* species in samples from healthy controls (Supplementary Fig. 1A). In general, the highest abundances of periodontal pathogens were observed in subgingival plaque samples, followed by saliva, and with the lowest abundance in tongue biofilm (Supplementary Fig. 1B).

### Global bacterial pathway expression is shaped mostly by site, but also by periodontitis

**Fig. 2:**
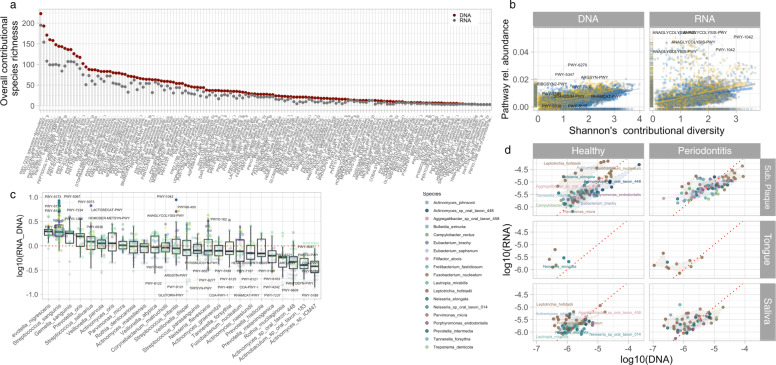
Pathway-level contributional diversity and species transcriptomal activity of oral microbiome. **a** Overall contributional species richness represented in metagenomic (DNA) and metatranscriptomic (RNA) pathway profiles determined across all paired samples. Only pathways expressed (RNA) by at least two species were plotted. **b** Relationship between pathway relative abundance and contributional species diversity (Shannon diversity index) in metagenomic (DNA) and metatranscriptomic (RNA). Each point represents a pathway and depicts its sample abundance as well species diversity (Shannon diversity index) potentially contributing (DNA) or actively contributing (RNA). We found that pathway abundance and contributional species diversity quite correlate in metagenomes (DNA) while some pathways clearly exhibited overexpression (RNA). Some highly expressed pathways which are not necessarily expressed by highly diverse communities. Pathway code was display for pathway with Abundance > 0.045 or shannons_div < 0.03. **c** Differences in per-pathway metagenomic (DNA) versus metatranscriptomic (RNA) contributions of top bacterial species as described in Fig. 1c. Each point represents the overall species transcriptional activity averaged within samples from different sites from the same Subject and across patients. Some species exhibited an overall tendency for overtranscription or undertranscription, whereas others displayed pathway-specific activity patterns. Boxplots display first quartile, median, and third quartile and whiskers represent 1.5 times the interquartile range from the first and third quartiles. **d** Pathway-level transcriptomal activity of species found to be differentially abundant between healthy and patients with periodontitis (see Supplementary Fig. 1A). The activity of each species is average within sample (average of pathways detected within paired metagenomes and metatranscriptomes).

Figure 2a shows overall pathway species contribution richness for both metagenomic (DNA) and metatranscriptomic (RNA) datasets in all samples. As can be seen, all pathways were not expressed (RNA) by all taxa with a potential of expressing the corresponding pathways (DNA). Some pathways, such as adenosine ribonucleotides de novo biosynthesis and guanosine ribonucleotides de novo biosynthesis, which are critical to cell survival, could be expressed by almost all members of the oral microbiota (>190 taxa). On the other hand, more specialized pathways involved in carbohydrate metabolism, including starch degradation V and lactose and galactose degradation I, could be expressed by less than 75 different taxa.

Figure 2b displays the relationship between pathway abundance and contributional species diversity (Shannon diversity index). Each point represents a specific pathway and depicts its sample abundance as well as diversity of species with potential (DNA) and actual (RNA) contribution. Correlation in terms of pathway abundance and contributing species diversity was observed in metagenomes (DNA). While the same trend was also observed in the metatranscriptomes (RNA), some pathways were clearly identified with high expression abundances despite low bacterial numbers of the contributing bacteria. In total, no differences were seen in overall bacterial activity (log10(RNA/DNA)) at each site in relation to health status. However, a significantly higher overall bacterial activity was observed in subgingival plaque samples as compared to saliva and tongue (Supplementary Fig. 2).

Figure 2c shows per-pathway DNA versus RNA contributions of top 27 bacterial species as described in Fig. 1c. Each point represents the overall species transcriptional activity averaged within samples from the three different sites in all individuals. Major differences were observed within each predominant genus, with some members exhibiting an overall tendency for high transcriptional activity (log10(RNA/DNA) > 0), as compared low (log10(RNA/DNA) < 0) by other members. In general, the highest RNA/DNA ratios were identified in *Streptococcus* and *Prevotella* species, while *Actinomyces* species in general were identified with (log10(RNA/DNA) < 0). As seen, high species-specific expression of certain pathways was observed, as demonstrated by *LACTOSECAT-PWY* and *ANAGLYCOLYSIS-PWY* by *Streptococcus salivarius* and *Streptococcus infantis*, respectively.

Figure 2d shows the pathway expression by bacterial transcriptional activities, of the 25 bacterial species identified with significantly different relative abundance in samples from patients with periodontitis versus healthy controls (Supplementary Fig. 1A). Bacterial expression profiles were heavily influenced not only by site but also by health status. For example, higher bacterial activity of *Leptotrichia hofstadii* was observed in both subgingival plaque and saliva collected from healthy controls, as compared to samples from patients with periodontitis.

### Significantly different bacterial pathway expression in subgingival plaque and saliva in patients with periodontitis

**Fig. 3:**
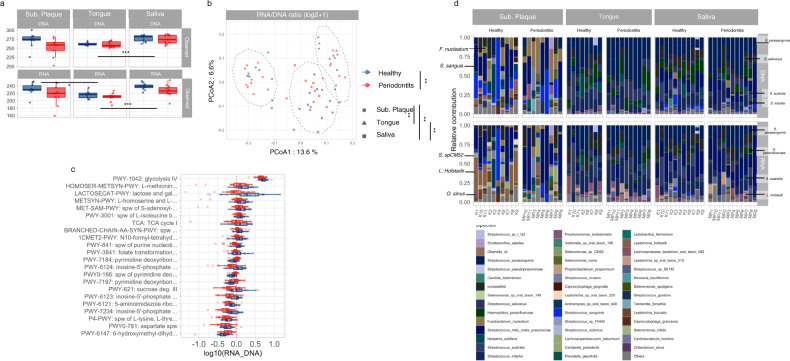
Pathway diversity, differential expression, and contributing species. **a** α-diversity patterns of potential (DNA) and expressed (RNA) pathways as measured by observed richness, Pielou’s evenness and Shannon diversity index. **b** β-diversity of pathway expression as expressed by log(RNA/DNA ratio) and summarized using weighted Jaccard distance and visualized using PCoA ordination. Significant differences were assessed using Kruskal–Wallis and PERMANOVAS tests for α and β-diversity, respectively. Significant FDR-adjusted *p* values were indicated as follows: *0.05 > *p* > 0.01 **0.01 > *p* > 0.001, ***p < 0.001. **c** Pathways exhibiting significant differential expression between healthy and periodontitis patients as identified using MaAsLin2. Color indicates heath status (blue: healthy controls versus red: periodontitis group), and shape reflects oral site. **d** Species relative contribution to the differentially expressed pathways (identified in **c**). Boxplots display first quartile, median, and third quartile and whiskers represent 1.5 times the interquartile range from the first and third quartiles.

While α-diversity metrics of potential and expressed pathways (DNA and RNA datasets, respectively) were not significantly different between site or health status (Supplementary Fig. 3A), both site and health status had a significant impact on β-diversity of pathway RNA/DNA expression (Fig. 3a, b). In addition, bacterial activity of 22 pathways (as quantified by pathway-level RNA/DNA ratio) was significantly different between periodontitis versus healthy controls, when comparing samples from all sites. Nineteen pathways were highly expressed in conditions of oral health, most of which were pathways contributing to carbohydrate metabolism. On the other hand, only three pathways were identified with higher RNA/DNA ratios in periodontitis, including pyrimidine deoxyribonucleotide phosphorylation and 6-hydroxymethyl dihydropterin diphosphate biosynthesis I (Fig. 3b).

Figure 3c shows bacterial species with the potential to express (DNA) the pathways identified with different expression in periodontitis versus health, including which bacterial species that were actually expressing the pathways (RNA). As seen, the bacterial signatures, in terms of DNA and RNA expression, were completely different based on site. Furthermore, it was evident that while some species despite high abundances of specific pathways within the metagenomes, were actually not contributing extensively to the total expression profiles, as evident from their low levels of concomitant pathway’s proportion in the RNA dataset. In addition, RNA expression of the 22 significant pathways was clearly different in subgingival plaque samples in health versus diseases, with much higher RNA expression by *L. hofstadii* in health. When comparing samples from each site based on health status, a total of 14 pathways were identified with significantly different RNA/DNA ratios in subgingival plaque, whereas a significantly higher RNA/DNA ratio of sucrose degradation III was observed in saliva of the healthy samples. No differences in RNA/DNA ratios were observed in tongue biofilm in health versus periodontitis (Supplementary Fig. 3B, C).

### Bacterial gene expression reveals impaired carbohydrate metabolism in periodontitis

**Fig. 4:**
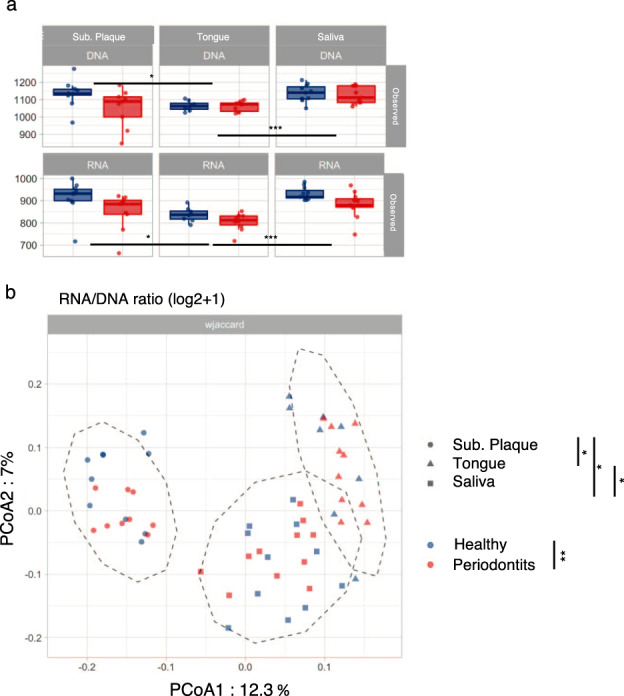
Diversity of microbial genes associated with associated with EC numbers. **a** α-diversity of gene families found in metagenomes (DNA) and metatranscriptomes (RNA) in plaque, tongue biofilm, and saliva expressed as number of observed gene families (i.e., richness). Boxplots display first quartile, median, and third quartile and whiskers represent 1.5 times the interquartile range from the first and third quartiles. **b** β-diversity of gene expression visualized as measured by log RNA/DNA ratio quantified using weighted Jaccard distance and visualized on PCoA. Sample denotation: red: periodontitis, blue: oral health, circle: plaque, triangle: tongue biofilm, square: saliva. Significant differences were assessed using Kruskal–Wallis and PERMANOVAS tests for α and β-diversity, respectively. Significant FDR-adjusted *p* values were indicated as follows: *0.05 > *p* > 0.01 **0.01 > *p* > 0.001 ***p < 0.001.

α-diversity of DNA and RNA gene expression as quantified by observed, evenness, and Shannon index was significantly influenced by site, but not by health status (Fig. 4a, Supplementary Fig. 4A). On the other hand, β-diversity of RNA/DNA ratios were significantly influenced by site, and also to a lesser but significant degree by health status (Fig. 4b, Supplementary Fig. 4B).

**Fig. 5:**
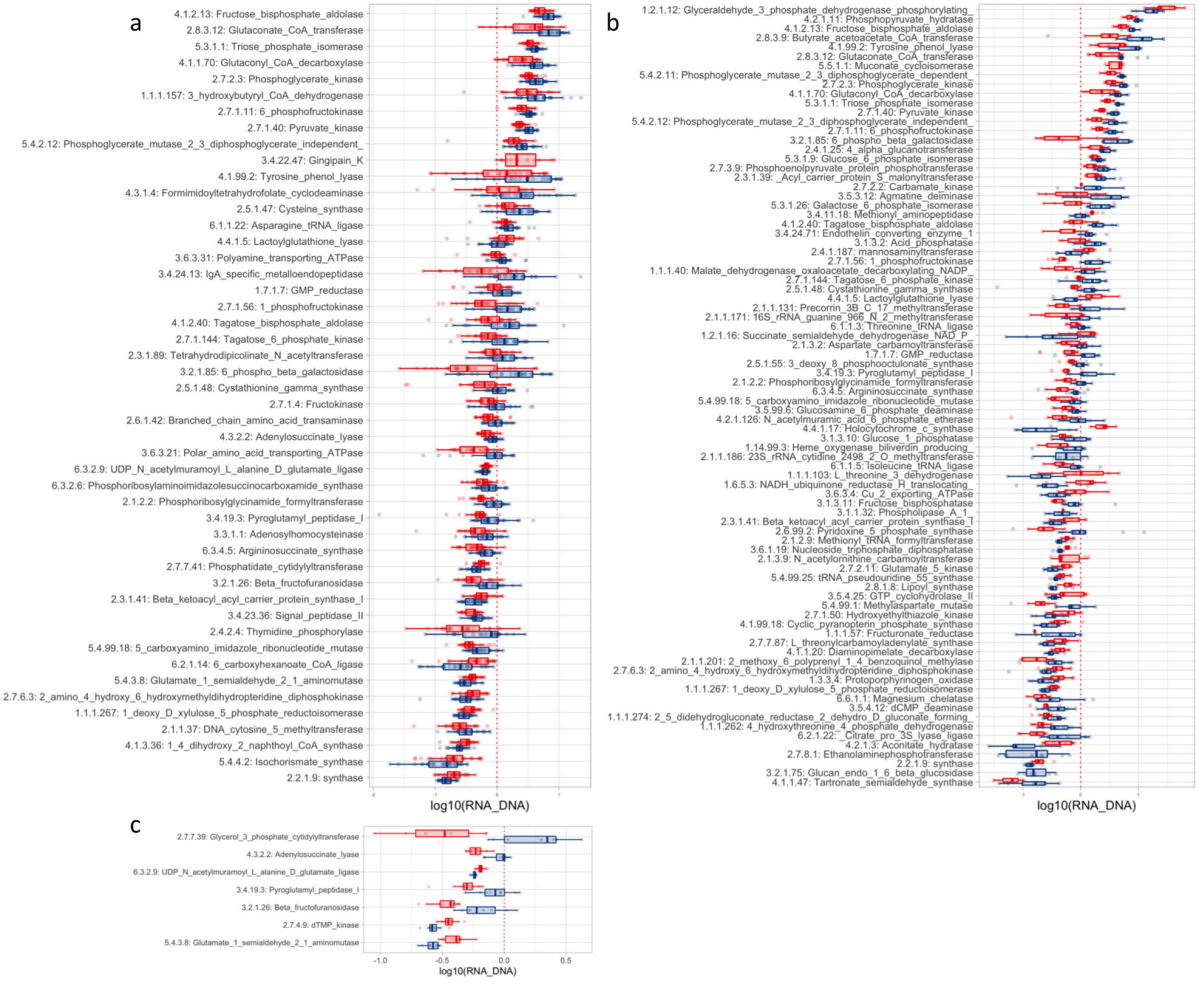
Genes (matching EC numbers) differentially expressed (RNA/DNA ratio) between healthy patient and patient with periodontal disease. **a** Overall, **b** plaque, and **c** tongue biofilm. No gene was found differentially expressed within saliva oral site. Boxplots display first quartile, median, and third quartile and whiskers represent 1.5 times the interquartile range from the first and third quartiles.

Figure 5a shows the 48 bacterial genes (EC numbers), which were identified by a significantly different RNA/DNA ratio in periodontitis versus health (based on analysis of all samples). Specifically, 36 genes were observed as having higher RNA/DNA ratios in health as compared to 12 genes in periodontitis. When performing pairwise comparison of samples collected at each site, 84 genes in subgingival plaque (Fig. 5b, 61 health associated, 23 periodontitis associated), and 7 genes in tongue (Figs. 5c, 4 health associated, 3 periodontitis associated), were identified with a significantly different RNA/DNA ratio in periodontitis versus health. No genes were observed with significant different RNA/DNA ratio in saliva samples obtained from health and periodontitis. Comparable findings were attained considering gene families matching KEGG’s orthologs (Supplementary Fig. 5A–D).

Nine out of the ten genes with the highest RNA/DNA ratios were significantly more expressed in oral health. Six of these genes contribute to carbohydrate metabolism, specifically in different steps of glycolysis. On the other hand, the only top ten gene identified with higher RNA/DNA ratio in periodontitis was *Gingipain_K*, which is solely expressed by the periodontal pathogen *Porphyromonas gingivalis* (Fig. 5a).

### Smoking has limited association with bacterial activity as compared to oral site and health status

The association of smoking was tested by comparison of pathway expression as determined by DNA, RNA, and RNA/DNA, respectively. Based on comparison of all 42 samples collected from smokers with that of 90 samples collected from nonsmokers, a total of four pathways were identified with significantly different DNA expression in samples from smokers (one with higher expression and three with lower expression), as compared to nonsmokers (Supplementary Fig. 6A). In addition, 12 pathways were recorded with significantly different RNA/DNA expression, with 9 of those pathways being more expressed in smokers (Supplementary Fig. 6B). No pathways were identified with significantly different RNA expression in smokers versus nonsmokers.

## Discussion

The purpose of the present investigation was to study the association of chronic inflammation, periodontitis, and bacterial gene expression profiles in three different compartments of the oral microbiota—subgingival plaque, tongue, and saliva. We used contemporary metagenomics and metatranscriptomics analyses to test the hypothesis that periodontitis associates with bacterial gene expression not only locally in the subgingival plaque microbiota but also in saliva and at distant sites such as the tongue.

As expected, major differences in taxonomic composition of predominant bacterial species identified in subgingival plaque, tongue biofilm, and saliva were observed (Fig. 1c). Indeed, this has been known since 2012, where 16S based analysis of samples from the Human Microbiome Project was used to characterize the composition of the microbiota at ten digestive tract sites^5^. Thus, our metagenomic data not only confirm previous findings from the Human Microbiome Project but the alignment of data also underline the robustness of the methods and databases used in the present study. While predominant bacterial species identified in subgingival plaque were almost completely absent in tongue, small amounts were identified in concomitant saliva samples. PCR and microarray techniques have been used to demonstrate concordance of specific bacterial species in subgingival plaque and saliva in patients with periodontitis^14,15^. Importantly, saliva is sterile, when entering the oral cavity^16^. Therefore, our species-resolution data analysis confirms the assumption of the salivary microbiota as a conglomerate of bacteria shed from various oral surfaces.

In health, substantial site-specific variations in taxonomic composition were observed within some genera, as exampled by major differences in relative abundance of specific *Rothia* and *Streptococcus* species identified in subgingival plaque versus tongue. Specifically, *Rothia dentocariosa* and *Streptococcus oralis* were identified with high abundances in subgingival plaque, but almost absent in tongue, whereas *Rothia mucilaginosa* and *S. salivarius* were predominant in tongue (Fig. 1c). While species within the same genus are taxonomically closely related, site-specific preferences support the idea of coevolution of the resident microbiota together with the host, which results in gradual adaptation of specific bacterial species to the environment offered at the various oral sites^17^.

Bacterial gene expression is shaped by oral site in health, as seen by significant differences in β-diversity at pathway (Fig. 3a) as well as gene level (Fig. 4b). Moreover, bacterial pathway expression as measured by their RNA/DNA ratios identified at each site in oral health demonstrated site-specific variations in term of general bacterial activity. This is clearly visualized in Fig. 2d with species such as *L. hofstadii* being placed in the upper left corner in subgingival plaque, in contrast to saliva and tongue. Likewise, species contribution of pathways being expressed significantly different in periodontitis and oral health were completely different at each site (Fig. 3c). Collectively, these findings confirm that taxonomic composition and bacterial gene expression of the resident, microbiota in oral health is shaped by the prevailing ecological properties, which are present at each niche in the oral cavity^18^.

Unsurprisingly, periodontitis was clearly associated with the composition of the subgingival plaque microbiota, as seen by significant different β-diversity (Fig. 1b), together with significantly higher relative abundance of predominant species such as *T. forsythia* (Fig. 1c). Also, 25 bacterial species were observed with higher abundances in subgingival plaque from patients with periodontitis (Supplementary Fig. 1A). Indeed, the composition of the subgingival microbiota of patients with periodontitis has for many decades been characterized intensively using a wide variety of culturing and molecular methods^19,20^. Collectively, these studies have identified the association of specific anaerobic gram-negative bacterial species with periodontitis, including the proposed periodontal pathogens, *P. gingivalis, T. denticola*, and *T. forsythia*, which was therefore named the red complex^21^. Indeed, some of the species we identified to associate with periodontitis, including *T. forsythia, F. alocis, P. micra, P. intermedia*, *T. denticola*, and *Fusobacterium nucleatum*, are consistently known in the literature as periodontal pathogens^1,22,23^. In addition, we identified several species, including *Bulleidia extructa*, *Campylobacter rectus*, *Desulfobulbus oralis*, and *Eubacterium* species, which have all previously been noted in the literature to potentially associate with periodontitis^24–27^. Thus, our data based on contemporary molecular methods not only reaffirm findings on a strong association of specific bacterial species with periodontitis but also underline the polymicrobial nature of the subgingival biofilm in periodontitis.

Notably, periodontitis was significantly associated with β-diversity of pathway and gene expression, as judged from their RNA/DNA ratios (Figs. S3A and 4A). Furthermore, pronounced differences were observed in specific bacterial gene expression in subgingival plaque at both pathway (Figs. 3a and S3B) and gene levels (Fig. 5a, b). Specifically, bacterial activity (RNA/DNA ratios) of pathways involved in carbohydrate metabolism, such as lactose and galactose degradation I and glucose and glucose-1 phosphate degradation, were significantly lower in subgingival plaque from patients with periodontitis. Also, significant differences were observed with genes involved in carbohydrate metabolism, where glyceraldehyde-3-phosphate dehydrogenase was significantly higher expressed in periodontitis, as compared to fructose bisphosphate aldolase, which was significantly less expressed. Multiple studies have used metagenomics to characterize the subgingival microbiota in periodontitis^22^, and data from these studies point toward a diseased microbiota, with increased gene expression of virulence factors involved in lipopolysaccharide synthesis and amino acid metabolism^22,28,29^. Indeed, our data reveal a decrease in carbohydrate metabolism, rather than increased expression of specific virulence factors. Nevertheless, data from our study and previous metagenomics reports probably reflect that the microbiota compositionally and functionally adapts to the ecological characteristics of periodontitis, such as conditions of anaerobiosis and chronic inflammation being present in the local periodontal environment^20,21^.

Historically, the role of the oral microbiota in the pathogenesis of periodontitis has been explained by different theories, i.e., the nonspecific plaque hypothesis, the specific plaque hypothesis, and the ecological plaque hypothesis^30^, which were then followed by the key stone pathogen hypothesis^31^, and finally the very recent inflammation-mediated polymicrobial-emergence and dysbiotic-exacerbation model^32^. Indeed, the focus of the nonspecific and the specific plaque hypothesis was primarily on the microbiota, as evaluated by the amount of biofilm and presence of specific pathogens, respectively^30^. On the contrary, the ecological plaque hypothesis was the first to pinpoint the important role of the interaction between the microbiota and the host in periodontitis^33^, which is the center of the inflammation-mediated polymicrobial-emergence and dysbiotic-exacerbation model^32^. Accordingly, the attention has gradually shifted from being much focused on presence of specific pathogens, to a more inclusive view, with emphasis on the activity of the biofilm, and how this is shaped by the conditions of chronic inflammation as coined by inflammation-mediated polymicrobial-emergence and dysbiotic-exacerbation model^32^. Importantly, this shift has only been possible with the advent of metatranscriptomics, which has enabled the possibility to characterize the functional activities, rather than just the taxonomic composition of the oral microbiota^34^. Consequently, our findings of impaired carbohydrate metabolism of the oral microbiota at multiple oral sites in periodontitis, which was most likely the consequence of the dysbiotic conditions determined by chronic periodontal inflammation, are in concert with the current view, stressing the interaction of the microbiota and the host as the determining factor in periodontitis, rather than solely the presence of specific proposed pathogens. Furthermore, taken together with a recent theory suggesting that frequent carbohydrate consumption may induce inflammation in the periodontal tissues^35^, our finding that periodontitis impairs carbohydrate metabolism of the subgingival plaque microbiota provides a possible explanation as to why excessive carbohydrate intake may contribute to the pathogenesis of periodontitis. Notably, periodontitis is linked with medical disorders such as type 2 diabetes, with conditions of systemic low-grade inflammation as the immediate communality^36^. Thus, it is an interesting hypothesis that impaired bacterial carbohydrate metabolism could be a factor aggravating systemic low-grade inflammation, in general.

Periodontitis was also associated with bacterial activity in saliva and the tongue, as visualized by specific pathways and their gene expression profiles (Figs. S3C and 5C). In addition, higher activity of specific bacterial species such as *L. hofstadii* was evident in saliva from healthy individuals (Fig. 2b). Specifically, significantly lower bacterial activity of the sucrose degradation III pathway was observed in saliva from patients with periodontitis. Furthermore, bacterial activity of genes related to lipid metabolism (glycerol-3-phosphate-cytidylyltransferase) and carbohydrate metabolism (β-fructofuranosidase) was significantly lower in tongue biofilm from patients with periodontitis. We have previously showed an impact of periodontitis on salivary bacterial activity^10^. However, this is the first study to perform simultaneous characterization of bacterial pathways and their gene expression profiles in subgingival plaque, tongue, and saliva. It is therefore interesting that pathways and genes identified with significantly different RNA/DNA ratios in saliva and tongue were not the same as those identified in subgingival plaque. Therefore, data suggest that periodontitis acts differently on bacterial gene expression in different oral compartments as visualized in the present study by subgingival plaque, tongue, and saliva.

Another interesting finding, which was evident from analysis of our high-resolution dataset, was that expression of *Gingipain_K* was solely identified in samples from patients with periodontitis (Fig. 5a). *Gingipain_K* is an endopeptidase with strict specificity for lysyl bonds, which is only produced by the periodontal pathogen *P. gingivalis*^37^. Interestingly, previous in vitro studies have shown that presence of *P. gingivalis* dramatically alters the transcriptomic profiles of oral commensals in an artificially grown biofilm^38^, which is one of the reasons that *P. gingivalis* is the main act in the key stone hypothesis^31^. Our finding of bacterial expression of *Gingipain_K* exclusively in samples from patients with periodontitis is therefore intriguing. However, future studies are warranted to reveal if *P. gingivalis* in vivo also alters the transcriptome of the resident microbiota or alternatively is counteracted by reliance mechanism of the commensals.

Some limitations apply to the present investigation, including the relatively small sample size, which however is comparable to other metatranscriptomic-based studies on the oral microbiota^39–41^. Furthermore, we pooled subgingival samples collected from the deepest periodontal pockets. Obviously, pooled subgingival plaque samples are not representative of specific subgingival sites^42^. In addition, samples were collected from both smokers and nonsmokers. Smoking has been demonstrated to profoundly impact the taxonomic composition of the subgingival microbiota^8,43,44^, whereas the effect on the salivary microbiota is probably less pronounced^45^. Therefore, we directly tested the association of smoking on DNA, RNA, and RNA/DNA expression (Supplementary Fig. 6A, B). Indeed, data showed limited association of smoking with these parameters, as compared to the main endpoints tested, oral site, and periodontitis. Nevertheless, future studies on bacterial activity should ideally be performed in nonsmokers. Finally, even though very deep sequencing of DNA and RNA was performed, it was still not possible to portray total gene expression of specific species with an overall low abundance. Accordingly, we could not determine if the transcriptomic profile of for example *P. gingivalis* differentiated between sites and in health versus disease.

In conclusion, data from the present study characterize the association of periodontitis with bacterial gene expression of the oral microbiota. Conditions of periodontitis was associated with bacterial activity of both subgingival plaque but also on tongue and the salivary microbiota. Collectively, data suggest that periodontitis associates with impaired carbohydrate metabolism of the oral microbiota. Future longitudinal and interventional studies are warranted to evaluate the potential pathogenic role of impaired bacterial carbohydrate metabolism not only in periodontitis but also in other diseases with low-grade inflammation, such as type 2 diabetes mellitus.

## Methods

### Study population and sample collection

The study population included 11 patients with chronic periodontitis, and 11 orally healthy controls, which were enrolled in January 2018 at University of Copenhagen, Department of Odontology. All 22 participants signed informed consent. The study was approved by the regional ethical committee (H-16016368) and reported to the local data authorization of University of Copenhagen (SUND-2018-8). Periodontitis was defined as bleeding on probing ≥25% of total sites + minimum two teeth with clinical attachment level ≥4 mm + minimum two teeth with probing depth ≥6 mm^46^. Exclusion criteria were: age <50 years, systemic diseases, and use of any kind of medication including usage of antibiotics within the last 3 months.

The periodontitis group was comprised of five males and six females with a mean age of 64 years (55–77 years), whereas the control group included four males and seven females with at mean age of 60 years (50–71 years). Four patients with periodontitis were current daily smokers, as compared to three current smokers in the control group. All 22 participants signed informed consent. The study was approved by the regional ethical committee (H-16016368) and reported to the local data authorization of University of Copenhagen (SUND-2018-8).

A total of three microbial samples were collected from each participants, including subgingival plaque samples (*n* = 22), tongue scrapings (*n* = 22), and stimulated saliva samples (*n* = 22), which were collected between 8:00 a.m. and 11:00 a.m. Microbial samples were consequently collected in the same order: stimulated saliva, tongue coating, and subgingival plaque according to standardized protocols^13,47,48^. Stimulated 1 mL of chewing-stimulated saliva was collected using paraffin gum. Subgingival plaque tongue biofilm was collected using a dental curette and a tongue spatula, respectively, and thereafter suspended in 2 mL sodium chloride (9 mg/mL). Immediately, after collection each sample was divided in two aliquots of 1 mL each, one each for metagenomics and metatranscriptomics analysis. RNAlater (Life Technologies, Denmark) was added to the aliquot allocated for metatranscriptomics and all aliquots were immediately stored at −80 °C until further processing.

### Metagenomic and metatranscriptomic library preparation and sequencing

Preparation of DNA and RNA library was performed according to Illumina’s TruSeq Nano DNA Sample Preparation Protocol and stranded mRNA, respectively. DNA samples were sheared on a Covaris E220 to ~450 bp, following the manufacturer’s recommendation, and uniquely tagged with one of Illumina’s TruSeq HT DNA dual barcode combination to enable sample pooling for sequencing. The following modifications were applied to Illumina’s TruSeq Stranded mRNA protocol. The oligo-dT mRNA purification step was omitted and instead, 200 ng of total RNA was directly added to the Elution2-Frag-Prime step. The PCR amplification step, which selectively enriches for library fragments that have adapters ligated on both ends, was performed according to the manufacturer’s recommendation but the number of amplification cycles was reduced to 12. Each library was uniquely tagged with one of Illumina’s TruSeq HT RNA dual barcode combination to allow pooling of libraries for sequencing. Both DNA and RNA libraries were quantitated using Promega’s QuantiFluor dsDNA assay and the average library size was determined on an Agilent Tapestation 4200. Library concentrations were then normalized to 4 nM and validated by qPCR on a QuantStudio-3 real-time PCR system (Applied Biosystems), using the Kapa library quantification kit for Illumina platforms (Kapa Biosystems).

The libraries were then pooled at equimolar concentrations and sequenced on the Illumina HiSeq2500 platform at a read length of 250 bp paired-end and 100 bp paired-end for DNA and RNA libraries, respectively.

### Read preprocessing

lllumina TruSeq adapters, 5′ or 3′ bases with quality scores lower than 20, as well as read pairs having a mate with any ambiguous base (i.e., N) or shorter than 150 or 50 bp for DNA and RNA sequences, respectively, were trimmed using Atropos^49^ in paired-end mode (version 1.1,25, -max-n 0 -n 1 -q 20,20 -quality-base 33 -minimum-length 150/50 -O 6).

RNA reads were then subjected to sortmeRNA^50^ version 2.1, default parameters, 5S, 16S, 23S, 18S, and 28S databases), using provided rRNA databases for in silico depletion of ribosomal RNA. Ribosomal RNA sequences represented an average of 83.9% of the quality-trimmed RNA reads (71.1–88.4%). Human reads were then removed from both metagenomic and metatranscriptomic datasets by aligning DNA and mRNA read pairs to the human genome (GRCh38, ftp://ftp.ebi.ac.uk/pub/databases/gencode/Gencode_human/release_28/GRCh38.primary_assembly.genome.fa.gz, downloaded on August 8, 2018). Alignments were performed using Bowtie2^51^ (version 2.3.4.1, -dovetail) for DNA reads and hisat2 (^52^version 2.1.0, -dta) for RNA reads, respectively. Samtools^53^ (version 1.3, view -b -f 12 -F 256) and bedtools^54^ (version 2.24.0, bamToFastq) were then used to identify and extract read pairs which consistently did not map the human reference genome, those were considered for microbial and taxonomical and functional profiling.

### Taxonomic and functional community profiling

Taxonomic composition of metagenomes was assessed using MetaPhlAn (version 3.0.1, June 25, 2020, -min_ab 0.000001 using the latest available database, i.e., mpa_v30_CHOCOPhlAn_201901). Briefly, MetaPhlan relies on read mapping against a built-in collection of clade-specific marker genes database which allows an unambiguous estimation of species relative abundance across samples with a low species-resolution false positives rate as compared to read classifiers. This is especially true when working with human microbiome data (https://www.researchgate.net/publication/343635031_Tutorial_Assessing_metagenomics_software_with_the_CAMI_benchmarking_toolkit). As the tool is based on marker genes and is not designed to assign taxonomic information to all reads nor rRNA sequence as taxonomic marker, a direct taxonomic classification of the metatranscriptomes is not possible.

Sample-specific taxonomic profiles generated using MetaPhlAn on metagenomes were used to filter the HUMAnN2^55^ built-in pangenomes database to the organism present in the sample. Metagenomic and metatranscriptomic reads were then mapped using Bowtie2 to sample-specific pangenomes. Metagenomic and metatranscriptomic reads failing to align to the pangenome databases were then blasted against UniRef90 using DIAMOND^56^. Hits were counted per gene family and normalized for length and alignment quality. Gene family abundances were then combined into pathways level using MetaCyc^57^ and normalized to relative abundances. Functional gene family tables were regrouped to KEGG’s orthologs and Enzyme (enzyme commission number, i.e., EC numbers) with the provided humann2 UniRef90_to_KO and UniRef90_to_EC numbers mapping files, respectively^55^.

Since sample-specific HUMAnN2 pangenome database are filtered based on MetaPhlAn taxonomic profiles, a complementary taxonomic profiling analysis was conducted using mOTUs profiler^58^ (version 2.5.0) which also includes MAGs (i.e., species without reference genomes in the standard databases) to confirm the observed patterns (Supplementary Fig. 7).

### Statistical analysis

Taxonomic profiles generated using MetaPhlAn as well as functional metagenomic and metatranscriptomic pathway and gene family tables generated using HUMAnN2 were imported in R and loaded into phyloseq objects for data handling^59^.

Taxonomic tables as well as pathway and gene family tables were characterized using α-diversity indices (i.e., number of observed species/pathways/gene families, Shannon diversity index, and Pielou’s evenness). Taxonomic and functional data were also characterized in terms of β-diversity (i.e., taxonomic community structure and metabolic profiles) using Atchinson distance computed on taxonomic count tables as well as weighted and binary Jaccard metrics for pathways and genes relative abundance normalized tables. Principle coordinate analysis (i.e., PCoA) was used to depict microbial community structure using vegan R package (*cmdscale()*). Relationship with community distance and ordination space was assessed using Shepard diagrams to ensure quality of the ordinations.

Differences between sites and health status were tested using two-sided nonparametric Kruskal–Wallis and PERMANOVA statistical tests for α and β-diversity, respectively. Multivariate Welch *t*-test was also run to confirm highlighted PERMANOVAS patterns^60^ and multivariate homogeneity was assessed to ensure PERMANOVA’s assumptions.

Aldex2 was used in order to detect differentially abundant species among oral site and health status on count normalized species tables^61^ and MaAsLin2 (https://huttenhower.sph.harvard.edu/maaslin/) for pathway and gene families relative abundance normalized tables. Pathway and gene families table from metagenomes (DNA) and metatranscriptomes (RNA) and computed DNA/RNA ratio were log transformed and only features occurring in at least 20% of the samples were considered. UNMAPPED and UNITEGRATED pathways were removed from α/β-diversity analysis but kept for linear modeling approach using MaAsLin2 in order to limit the potential for housekeeping functions to artificially inflate in less well-characterized samples.

When testing for differences between oral sites, subject identifier was added as random factor and PERMANOVA’s permutations were restricted to take into account repetitive sampling within the same Subject.

In order to correct for multiple comparisons *p* values generated using Kruskal–Wallis, Aldex2 Maaslin2 and PERMANOVAS tests were FDR corrected.

### Reporting summary

Further information on research design is available in the Nature Research Reporting Summary linked to this article.

## Supplementary information

Supplementary Information

Reporting Summary

## Data Availability

Raw sequence data have been deposited at NCBI (Sequence Read Archive) and are available under the BioProject PRJNA678453.

## Code availability

Bioinformatic open-source tools and parameters used in this present study are defined in the “Methods” section and scripts can be found in the following Github repository: https://github.com/fconstancias/oral-microbiome/. More details regarding the code to reproduce the analyses are available upon request.
