# Supplementary material for: Periodontitis associates with species-specific gene expression of the oral microbiota
Source: NPJ Biofilms Microbiomes. 2021 Sep 23;7:76. doi: 10.1038/s41522-021-00247-y (PMC8460658; doi:10.1038/s41522-021-00247-y)
Supplement: Supplementary file 1 — Supplementary Information [file 41522_2021_247_MOESM1_ESM.pdf]

Supplementary Figures:

a

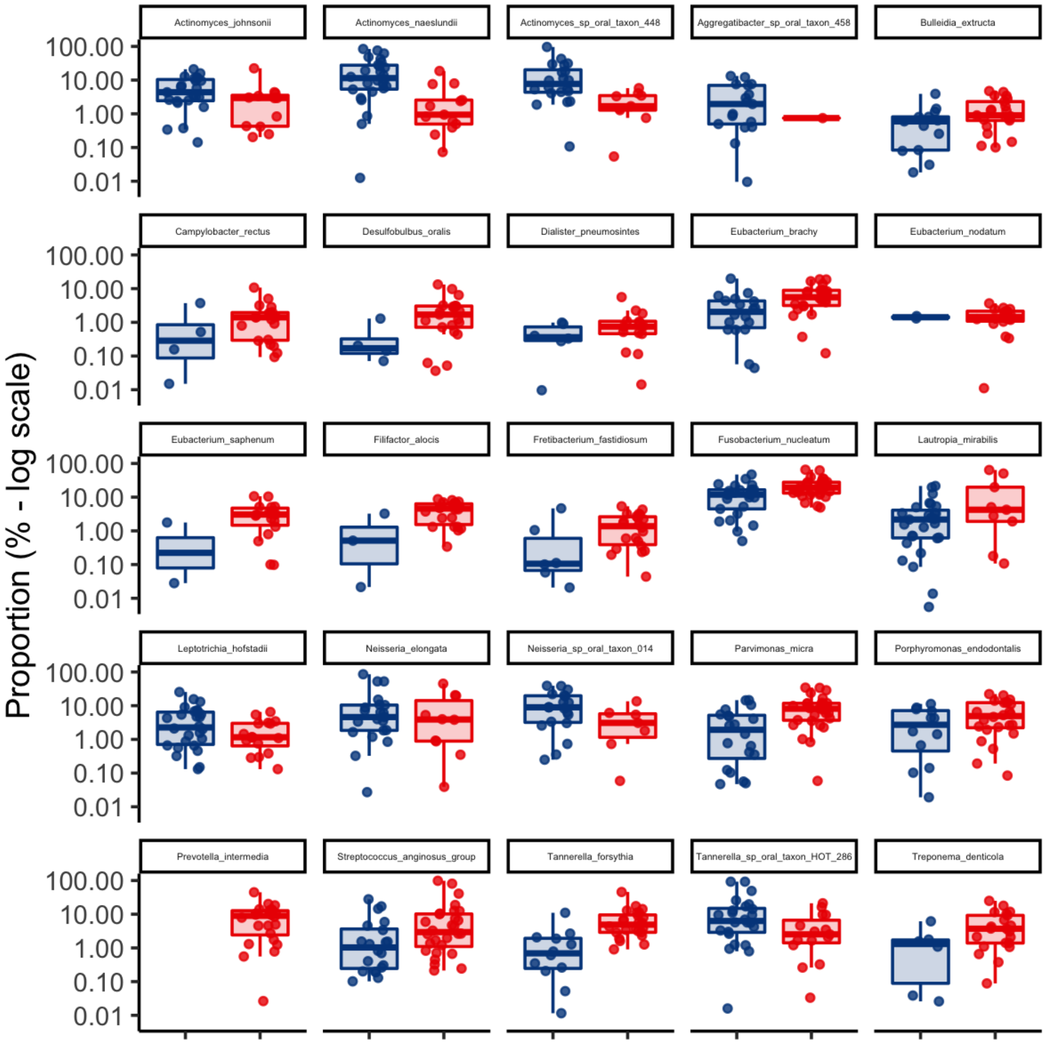

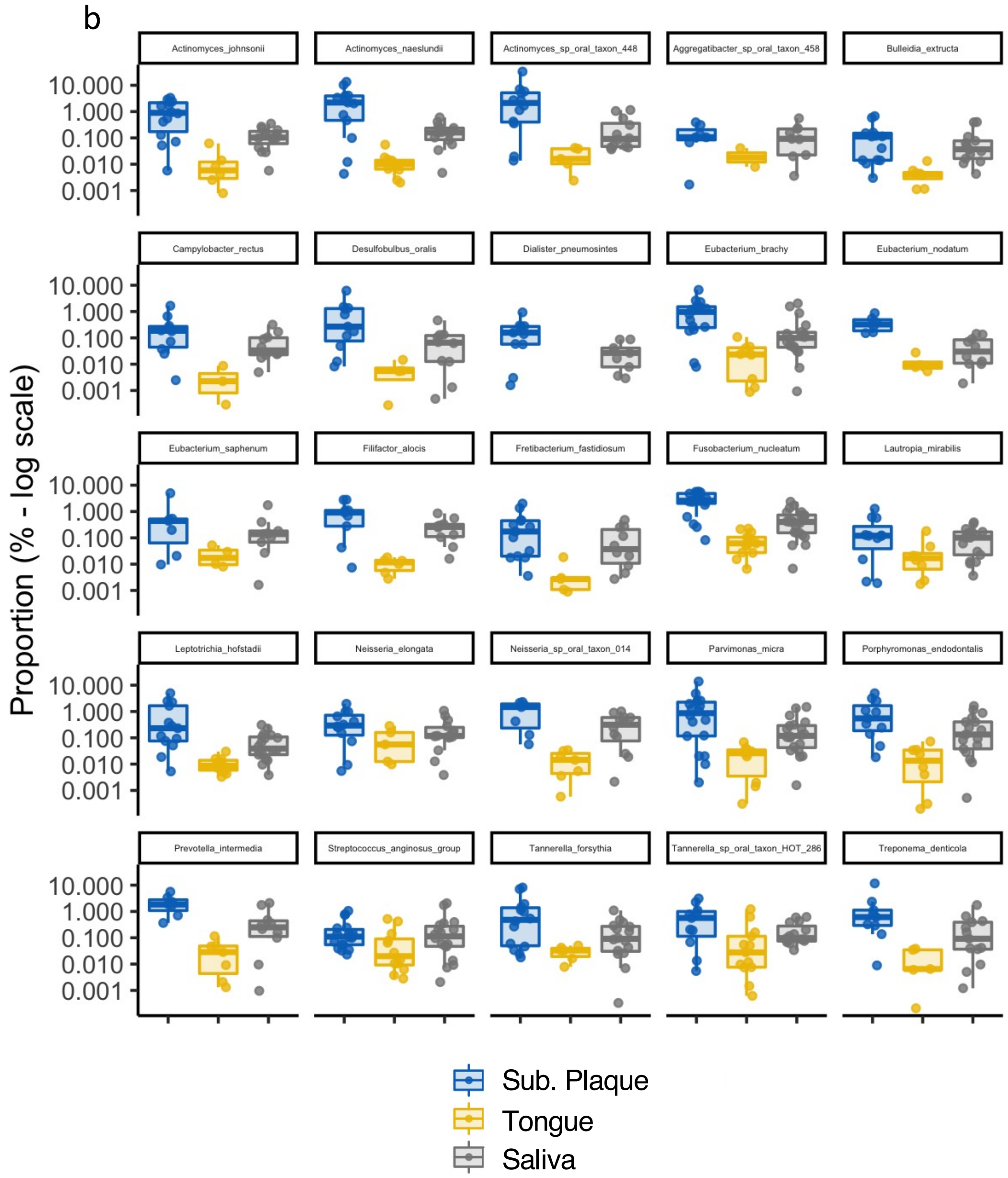

Fig. S1. <u>Species differentially abundant between:</u> A) healthy patients and patients with periodontitis disease. **B)** oral sites. Statistical tests were run on all the samples. Sample denotation: Red: periodontitis, dark blue: oral health, light blue: plaque, yellow: tongue and grey: saliva. Boxplots display first quartile, median, third quartile and whiskers represent 1.5 times the interquartile range from the first and third quartiles. Microbial taxonomy based on metaphlan v3.0.1 - default – ALDEx2::aldex.kw, denom="iqlr”. Only significant results are displayed FDRA adjusted p. value <0.05.

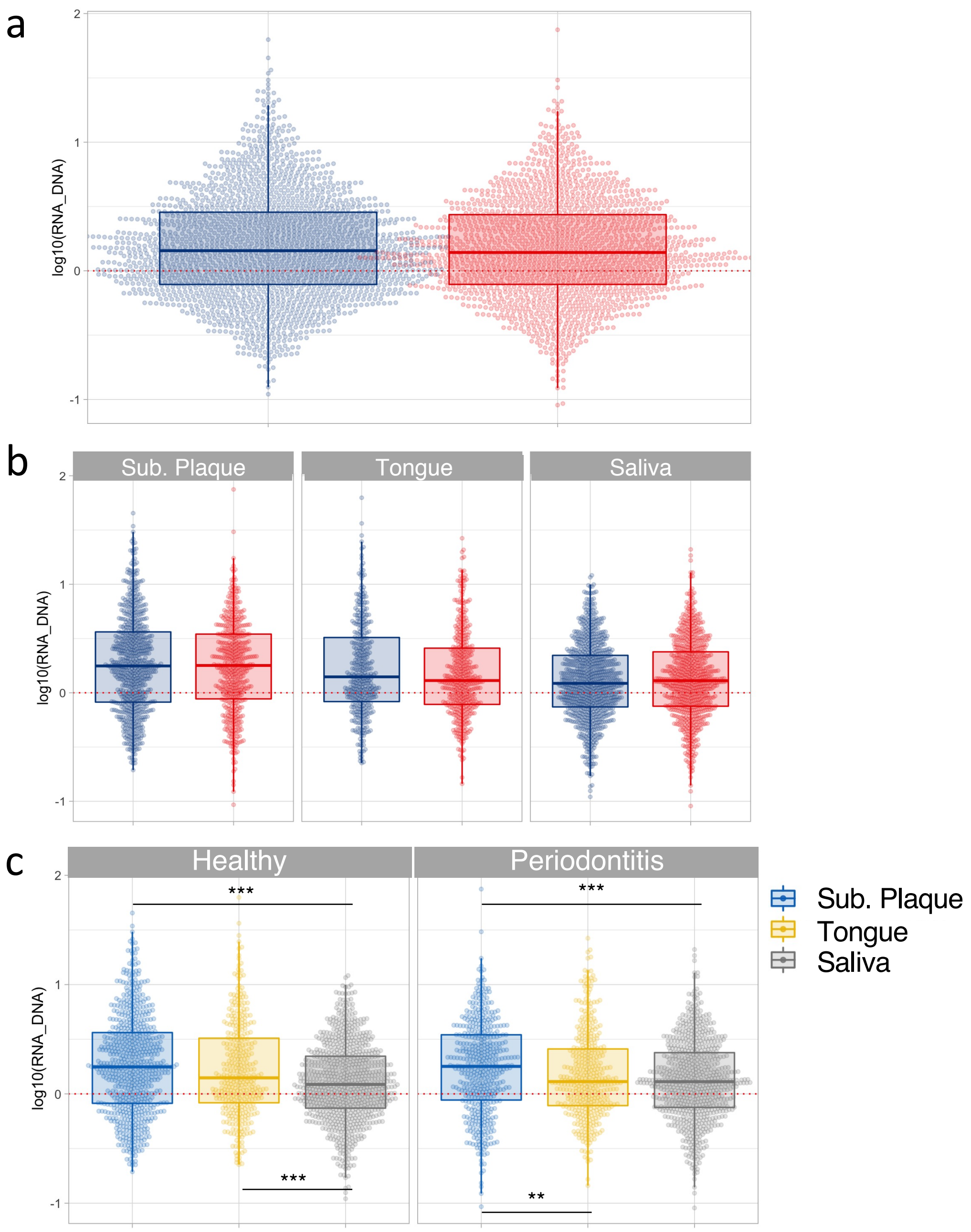

Fig. S2. <u>Pathway-level species metatranscriptomic activity:</u>

A) healthy patients and patients with periodontitis disease - overall, **B)** healthy patients and patients with periodontitis disease on each site, and **C)** among the different oral sites in healthy patients and patients with periodontitis disease. Each point represents the pathway transcriptional activity as measured by log10(RNA/DNA). Sample denotation: Red: periodontitis, dark blue: oral health, light blue: plaque, yellow: tongue and grey: saliva. Boxplots display first quartile, median, third quartile and whiskers represent 1.5 times the interquartile range from the first and third quartiles. Significant differences were determined using Kruskall-Wallis tests. Significant FDR-adjusted p-values were indicated as followed. * 0.05<p<0.01 ** 0.01 <p<0.001 *** p<0.001.

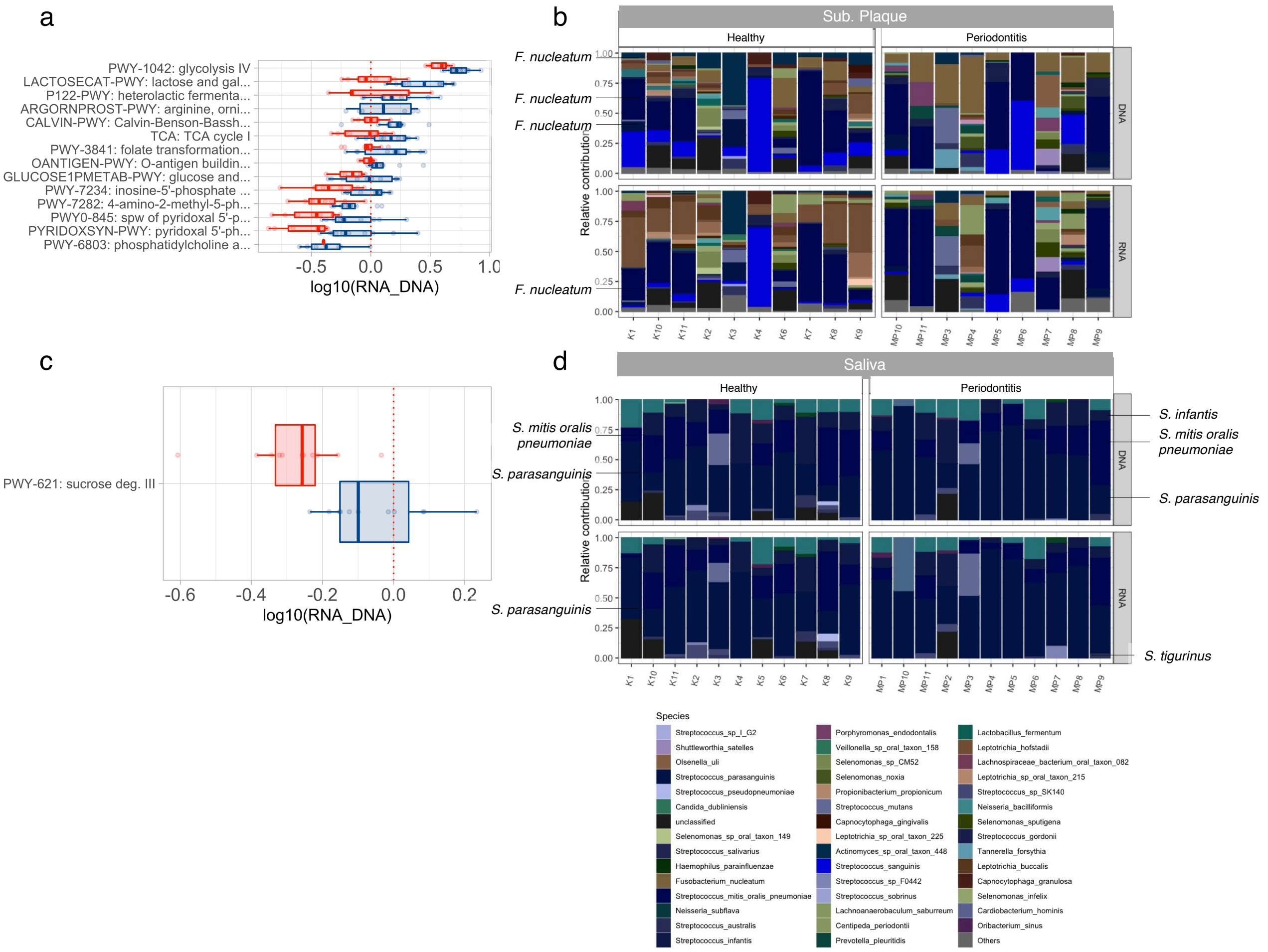

Fig. S3. <u>Differentially expressed pathways and contributing species in Plaque and Saliva sites:</u> Pathways exhibiting significant differential expression between healthy and periodontitis patients as identified using MaAsLin2 in **A)** plaque and **C)** saliva site. Colour indicates heath status (blue: healthy controls vs red: periodontitis group), and shape reflects oral site. Species relative contribution to the differentially expressed pathways (identified in Fig. S3C) in **B)** plaque and **D)** saliva. Species are coloured similarly as in Fig. 3. Boxplots display first quartile, median, third quartile and whiskers represent 1.5 times the interquartile range from the first and third quartiles.

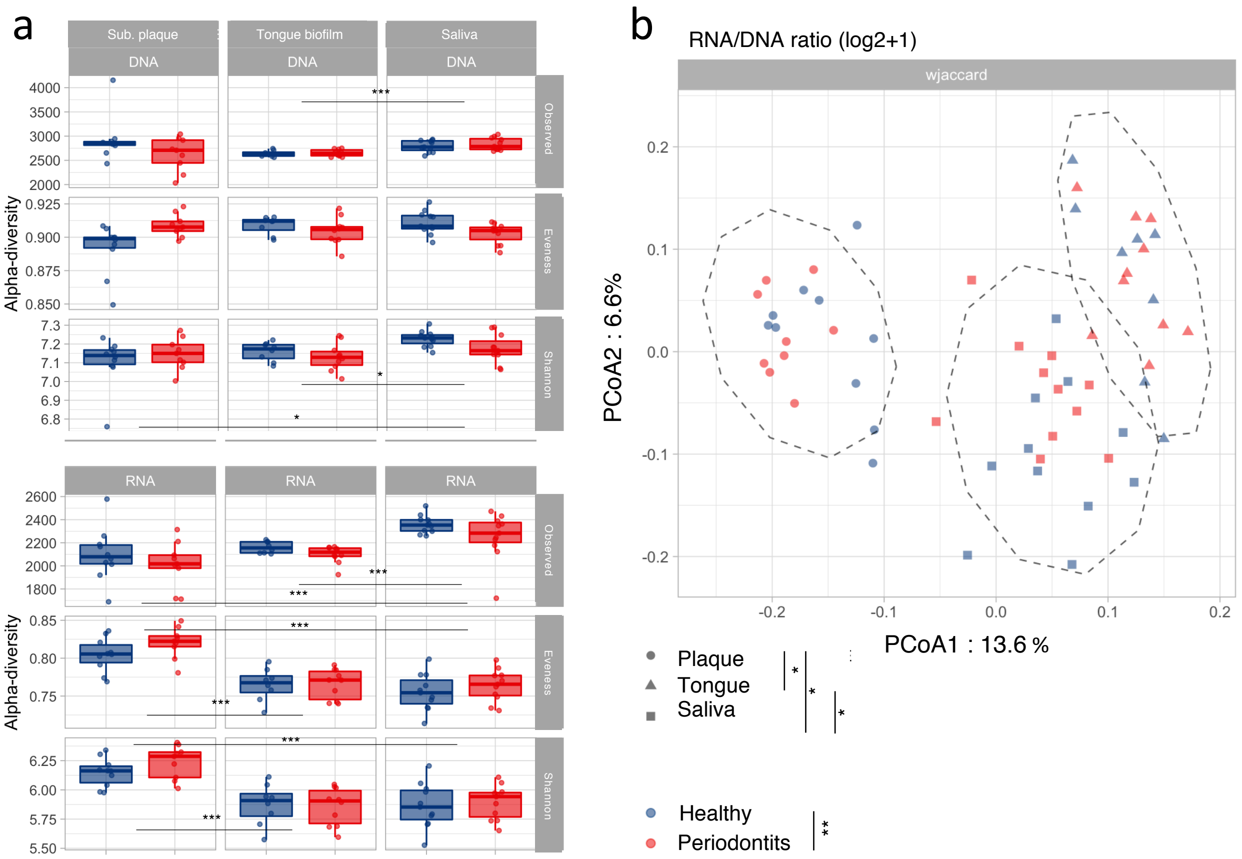

Fig S4. Diversity of microbial genes associated with associated with KEGG's Orthologs : **A)** Alpha-diversity of gene families found in metagenomes (DNA) and metatranscriptomes (RNA) in plaque, tongue biofilm and saliva expressed as number of Observed gene families (i.e., richness), Evenness and Shannon's diversity index. Boxplots display first quartile, median, third quartile and whiskers represent 1.5 times the interquartile range from the first and third quartiles. **B)** Beta diversity of gene expression visualized as measured by log RNA/DNA ratio quantified using weighted Jaccard distance and visualized on PCoA. Sample denotation: Red: periodontitis, Blue: oral health, Circle: plaque, Triangle: tongue biofilm, Square: saliva. Significant differencies were assessed using Kruskall-Wallis and PERMANOVAS tests for alpha and beta-diversity, respectively. Significant FDR-adjusted p-values were indicated as followed : * 0.05>p>0.01 ** 0.01>p>0.001 *** p<0.001.

a

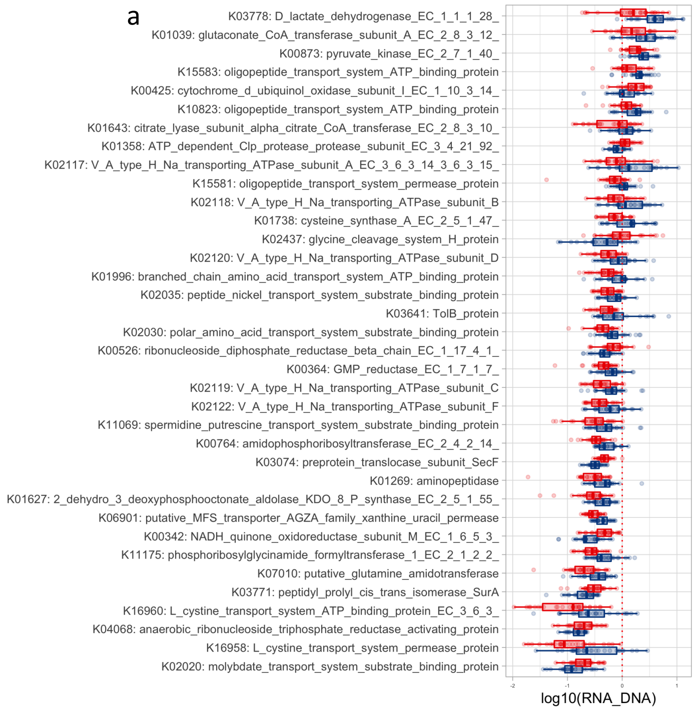
K03778: D_lactate_dehydrogenase_EC_1_1_1_28_
K01039: glutaconate_CoA_transferase_subunit_A_EC_2_8_3_12_
K00873: pyruvate_kinase_EC_2_7_1_40_
K15583: oligopeptide_transport_system_ATP_binding_protein
K00425: cytochrome_d_ubiquinol_oxidase_subunit_I_EC_1_10_3_14_
K10823: oligopeptide_transport_system_ATP_binding_protein
K01643: citrate_lyase_subunit_alpha_citrate_CoA_transferase_EC_2_8_3_10_
K01358: ATP_dependent_Clp_protease_protease_subunit_EC_3_4_21_92_
K02117: V_A_type_H_Na_transporting_ATPase_subunit_A_EC_3_6_3_14_3_6_3_15_
K15581: oligopeptide_transport_system_permease_protein
K02118: V_A_type_H_Na_transporting_ATPase_subunit_B
K01738: cysteine_synthase_A_EC_2_5_1_47_
K02437: glycine_cleavage_system_H_protein
K02120: V_A_type_H_Na_transporting_ATPase_subunit_D
K01996: branched_chain_amino_acid_transport_system_ATP_binding_protein
K02035: peptide_nickel_transport_system_substrate_binding_protein
K03641: TolB_protein
K02030: polar_amino_acid_transport_system_substrate_binding_protein
K00526: ribonucleoside_diphosphate_reductase_beta_chain_EC_1_17_4_1_
K00364: GMP_reductase_EC_1_7_1_7_
K02119: V_A_type_H_Na_transporting_ATPase_subunit_C
K02122: V_A_type_H_Na_transporting_ATPase_subunit_F
K11069: spermidine_putrescine_transport_system_substrate_binding_protein
K00764: amidophosphoribosyltransferase_EC_2_4_2_14_
K03074: preprotein_translocase_subunit_SecF
K01269: aminopeptidase
K01627: 2_dehydro_3_deoxyphosphooctonate_aldolase_KDO_8_P_synthase_EC_2_5_1_55_
K06901: putative_MFS_transporter_AGZA_family_xanthine_uracil_permease
K00342: NADH_quinone_oxidoreductase_subunit_M_EC_1_6_5_3_
K11175: phosphoribosylglycinamide_formyltransferase_1_EC_2_1_2_2_
K07010: putative_glutamine_amidotransferase
K03771: peptidyl_prolyl_cis_trans_isomerase_SurA
K16960: L_cystine_transport_system_ATP_binding_protein_EC_3_6_3_
K04068: anaerobic_ribonucleoside_triphosphate_reductase_activating_protein
K16958: L_cystine_transport_system_permease_protein
K02020: molybdate_transport_system_substrate_binding_protein
-2
-1
0
1
log10(RNA_DNA)

b

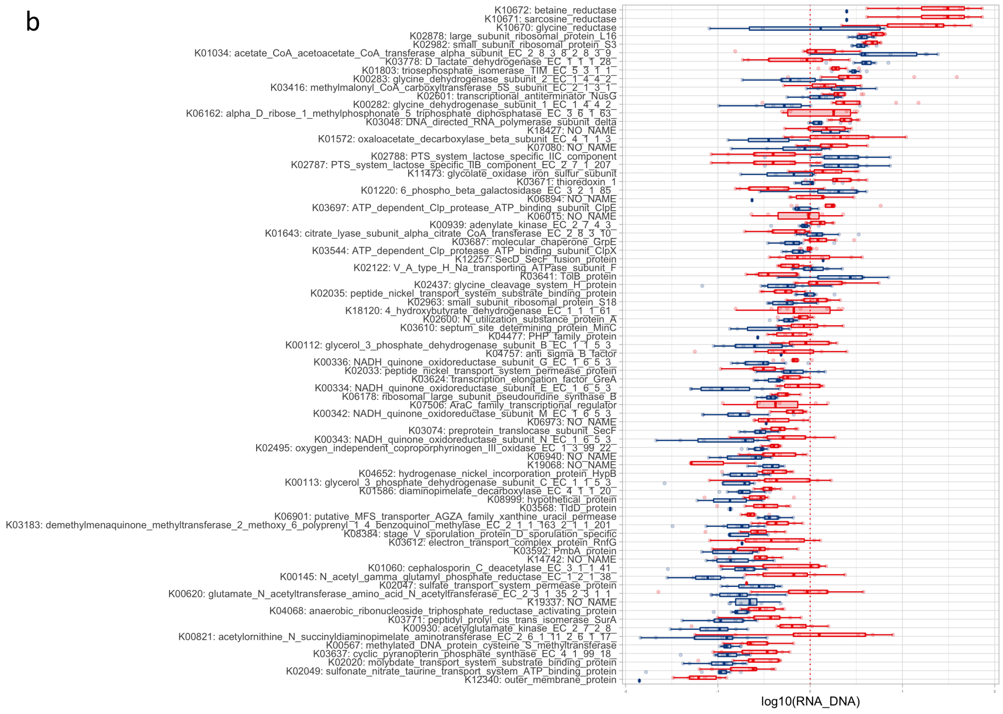
K10672: betaine_reductase
K10671: sarcosine_reductase
K10670: glycine_reductase
K02878: large_subunit_ribosomal_protein_L16
K02982: small_subunit_ribosomal_protein_S3
K01034: acetate_CoA_acetoacetate_CoA_transferase_alpha_subunit_EC_2_8_3_8_2_8_3_9_
K03778: D_lactate_dehydrogenase_EC_1_1_1_28_
K01803: triosephosphate_isomerase_TIM_EC_5_3_1_1_
K00283: glycine_dehydrogenase_subunit_2_EC_1_4_4_2_
K03416: methylmalonyl_CoA_carboxyltransferase_5S_subunit_EC_2_1_3_1_
K02601: transcriptional_antiterminator_NusG
K00282: glycine_dehydrogenase_subunit_1_EC_1_4_4_2_
K06162: alpha_D_ribose_1_methylphosphonate_5_triphosphate_diphosphatase_EC_3_6_1_63_
K03048: DNA_directed_RNA_polymerase_subunit_delta
K18427: NO_NAME
K01572: oxaloacetate_decarboxylase_beta_subunit_EC_4_1_1_3_
K07080: NO_NAME
K02788: PTS_system_lactose_specific_IIC_component
K02787: PTS_system_lactose_specific_IIB_component_EC_2_7_1_207_
K11473: glycolate_oxidase_iron_sulfur_subunit
K03671: thioredoxin_1
K01220: 6_phospho_beta_galactosidase_EC_3_2_1_85_
K06894: NO_NAME
K03697: ATP_dependent_Clp_protease_ATP_binding_subunit_ClpE
K06015: NO_NAME
K00939: adenylate_kinase_EC_2_7_4_3_
K01643: citrate_lyase_subunit_alpha_citrate_CoA_transferase_EC_2_8_3_10_
K03687: molecular_chaperone_GrpE
K03544: ATP_dependent_Clp_protease_ATP_binding_subunit_ClpX
K12257: SecD_SecF_fusion_protein
K02122: V_A_type_H_Na_transporting_ATPase_subunit_F
K03641: TolB_protein
K02437: glycine_cleavage_system_H_protein
K02035: peptide_nickel_transport_system_substrate_binding_protein
K02963: small_subunit_ribosomal_protein_S18
K18120: 4_hydroxybutyrate_dehydrogenase_EC_1_1_1_61_
K02600: N_utilization_substance_protein_A
K03610: septum_site_determining_protein_MinC
K04477: PHP_family_protein
K00112: glycerol_3_phosphate_dehydrogenase_subunit_B_EC_1_1_5_3_
K04757: anti_sigma_B_factor
K00336: NADH_quinone_oxidoreductase_subunit_G_EC_1_6_5_3_
K02033: peptide_nickel_transport_system_permease_protein
K03624: transcription_elongation_factor_GreA
K00334: NADH_quinone_oxidoreductase_subunit_E_EC_1_6_5_3_
K06178: ribosomal_large_subunit_pseudouridine_synthase_B
K07506: AraC_family_transcriptional_regulator
K00342: NADH_quinone_oxidoreductase_subunit_M_EC_1_6_5_3_
K06973: NO_NAME
K03074: preprotein_translocase_subunit_SecF
K00343: NADH_quinone_oxidoreductase_subunit_N_EC_1_6_5_3_
K02495: oxygen_independent_coproporphyrinogen_III_oxidase_EC_1_3_99_22_
K06940: NO_NAME
K19068: NO_NAME
K04652: hydrogenase_nickel_incorporation_protein_HypB
K00113: glycerol_3_phosphate_dehydrogenase_subunit_C_EC_1_1_5_3_
K01586: diaminopimelate_decarboxylase_EC_4_1_1_20_
K08999: hypothetical_protein
K03568: TldD_protein
K06901: putative_MFS_transporter_AGZA_family_xanthine_uracil_permease
K03183: demethylmenaquinone_methyltransferase_2_methoxy_6_polyprenyl_1_4_benzoquinol_methylase_EC_2_1_1_163_2_1_1_201_
K08384: stage_V_sporulation_protein_D_sporulation_specific
K03612: electron_transport_complex_protein_RnfG
K03592: PmbA_protein
K14742: NO_NAME
K01060: cephalosporin_C_deacetylase_EC_3_1_1_41_
K00145: N_acetyl_gamma_glutamyl_phosphate_reductase_EC_1_2_1_38_
K02047: sulfate_transport_system_permease_protein
K00620: glutamate_N_acetyltransferase_amino_acid_N_acetyltransferase_EC_2_3_1_35_2_3_1_1_
K19337: NO_NAME
K04068: anaerobic_ribonucleoside_triphosphate_reductase_activating_protein
K03771: peptidyl_prolyl_cis_trans_isomerase_SurA
K00930: acetylglutamate_kinase_EC_2_7_2_8_
K00821: acetylornithine_N_succinyldiaminopimelate_aminotransferase_EC_2_6_1_11_2_6_1_17_
K00567: methylated_DNA_protein_cysteine_S_methyltransferase
K03637: cyclic_pyranopterin_phosphate_synthase_EC_4_1_99_18_
K02020: molybdate_transport_system_substrate_binding_protein
K02049: sulfonate_nitrate_taurine_transport_system_ATP_binding_protein
K12340: outer_membrane_protein
log10(RNA_DNA)

c

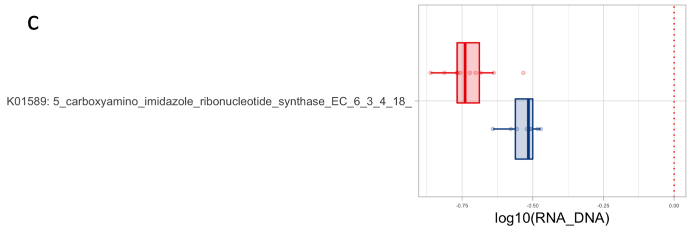
K01589: 5_carboxyamino_imidazole_ribonucleotide_synthase_EC_6_3_4_18_
-0.75
-0.50
-0.25
0.00
log10(RNA_DNA)

d

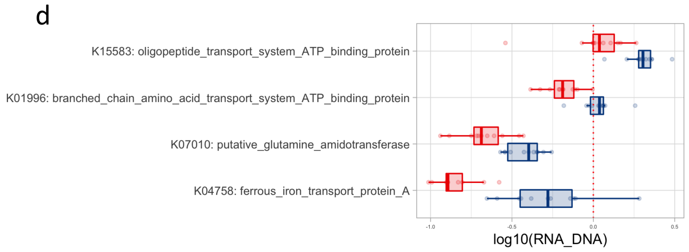

Fig S5. <u>Genes (mapping KEGG's orthologs) differentially expressed (RNA/DNA ratio) between healthy patient and patient with periodontal diseases:</u> A) overall, **B)** plaque, **C)** saliva and **D)** tongue site. Boxplots display first quartile, median, third quartile and whiskers represent 1.5 times the interquartile range from the first and third quartiles.

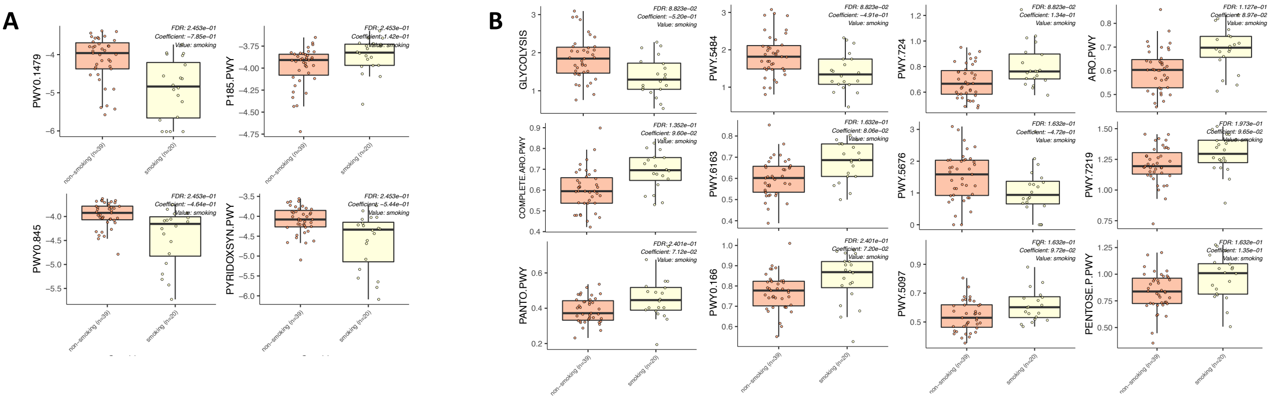

Fig S6. Pathway significantly associated with smoking status in terms of functional potential (A - DNA) and transcriptional activity (B – RNA/DNA) identified using MaAsLin2.

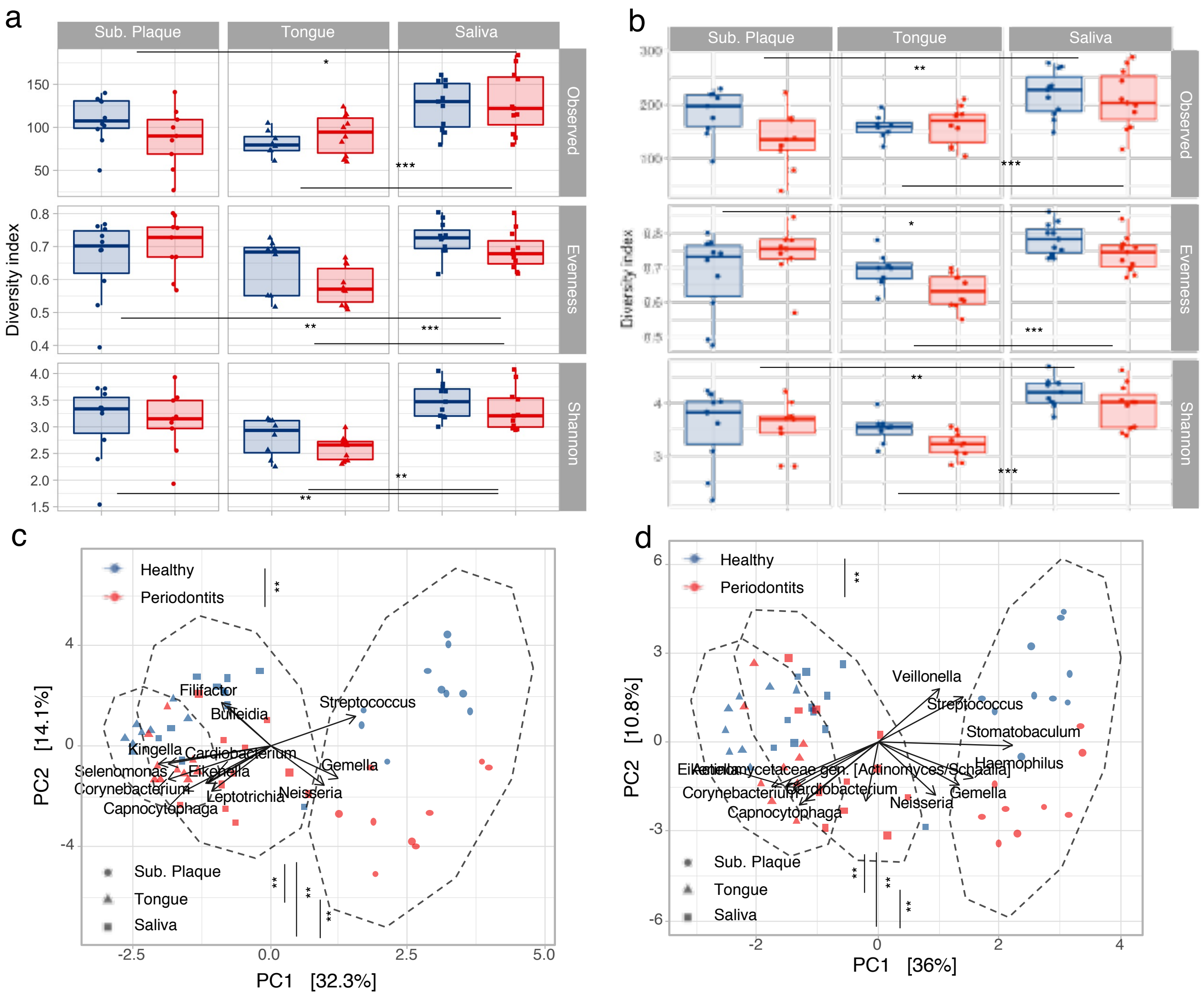

FigS7. Alpha and beta diversity of Oral microbiota characterized using metaphlan3 (a & c) and mOTUs (b & d) taxonomic profilers. a-b: α-diversity patterns as measured by Observed Richness, Pielou's Evenness and Shannon diversity index, **c-d** β-diversity as summarised by Aitchison distance and visualized using PCoA ordination. Colour indicates heath status (blue: healthy controls vs red: periodontitis group), and shape reflects oral site. Significant differences were assessed using Kruskall-Wallis and PERMANOVAS tests for alpha and beta-diversity, respectively. Significant FDR-adjusted p-values were indicated as followed : * 0.05>p>0.01 ** 0.01>p>0.001 *** p<0.001. Boxplots display first quartile, median, third quartile and whiskers represent 1.5 times the interquartile range from the first and third quartiles. The top 12 genus significantly (p<0.05) correlated among the PCoA 1 & 2 axes were displayed using vegan::envfit() function.
