# Supplementary material for: Periodontitis associates with species-specific gene expression of the oral microbiota
Source: NPJ Biofilms Microbiomes. 2021 Sep 23;7:76. doi: 10.1038/s41522-021-00247-y (PMC8460658; doi:10.1038/s41522-021-00247-y)
Supplement: Supplementary file 2 — Reporting Summary [file 41522_2021_247_MOESM2_ESM.pdf]

nature research

Corresponding author(s): Florentin CONSTANCIAS, Daniel BELSTRØM

Last updated by author(s): Aug 4, 2021

# Reporting Summary

Nature Research wishes to improve the reproducibility of the work that we publish. This form provides structure for consistency and transparency in reporting. For further information on Nature Research policies, see our Editorial Policies and the Editorial Policy Checklist.

## Statistics

For all statistical analyses, confirm that the following items are present in the figure legend, table legend, main text, or Methods section.

| n/a | Confirmed | |
|---|---|---|
| ☐ | ☒ | The exact sample size ($n$) for each experimental group/condition, given as a discrete number and unit of measurement |
| ☐ | ☒ | A statement on whether measurements were taken from distinct samples or whether the same sample was measured repeatedly |
| ☐ | ☒ | The statistical test(s) used AND whether they are one- or two-sided<br>*Only common tests should be described solely by name; describe more complex techniques in the Methods section.* |
| ☐ | ☒ | A description of all covariates tested |
| ☐ | ☒ | A description of any assumptions or corrections, such as tests of normality and adjustment for multiple comparisons |
| ☐ | ☒ | A full description of the statistical parameters including central tendency (e.g. means) or other basic estimates (e.g. regression coefficient) AND variation (e.g. standard deviation) or associated estimates of uncertainty (e.g. confidence intervals) |
| ☐ | ☒ | For null hypothesis testing, the test statistic (e.g. $F$, $t$, $r$) with confidence intervals, effect sizes, degrees of freedom and $P$ value noted<br>*Give P values as exact values whenever suitable.* |
| ☒ | ☐ | For Bayesian analysis, information on the choice of priors and Markov chain Monte Carlo settings |
| ☒ | ☐ | For hierarchical and complex designs, identification of the appropriate level for tests and full reporting of outcomes |
| ☒ | ☐ | Estimates of effect sizes (e.g. Cohen's $d$, Pearson's $r$), indicating how they were calculated |

*Our web collection on statistics for biologists contains articles on many of the points above.*

## Software and code

Policy information about availability of computer code

| | |
|---|---|
| Data collection | *Provide a description of all commercial, open source and custom code used to collect the data in this study, specifying the version used OR state that no software was used.* |
| Data analysis | Bioinformatic open-source tools and parameters used in this present study are defined in the respective method section of the manuscript. Code to reproduce the analyses is available upon reasonable request. |

For manuscripts utilizing custom algorithms or software that are central to the research but not yet described in published literature, software must be made available to editors and reviewers. We strongly encourage code deposition in a community repository (e.g. GitHub). See the Nature Research guidelines for submitting code & software for further information.

## Data

Policy information about availability of data

All manuscripts must include a data availability statement. This statement should provide the following information, where applicable:

- Accession codes, unique identifiers, or web links for publicly available datasets
- A list of figures that have associated raw data
- A description of any restrictions on data availability

Raw sequence data has been deposited in GenBank (Sequence Read Archive) and is available under the BioProject (-submission process in progress-).



April 2020

# Field-specific reporting

Please select the one below that is the best fit for your research. If you are not sure, read the appropriate sections before making your selection.

☒ Life sciences ☐ Behavioural & social sciences ☐ Ecological, evolutionary & environmental sciences

For a reference copy of the document with all sections, see nature.com/documents/nr-reporting-summary-flat.pdf

# Life sciences study design

All studies must disclose on these points even when the disclosure is negative.

| | |
|---|---|
| Sample size | The sample size was based on data from a previous study, in which we performed metagenomic and metatracriptomic characterization of the the salivary microbiota in patients with periodontitis, patients with dental caries and orally healthy controls (1). Accordingly, a sample size of n=10 in each group was sufficient to identify differences in taxonomic profiles as well as bacterial gene expression saliva.<br><br>1. Belstrom D et. al. Metagenomic and metatranscriptomic analysis of saliva reveals disease-associated microbiota in patients with periodontitis and dental caries. NPJ Biofilms Microbiomes. 2017 Oct 2;3:23. doi: 10.1038/s41522-017-0031-4. eCollection 2017. |
| Data exclusions | Patients with periodontitis were excluded if they had any other oral disease. Orally healthy controls were excluded if they had any oral disease. All participants were excluded if they had any systemic disease, and if they used any prescription medication, including use of systemic antibiotics within the latest three months prior to sampling. |
| Replication | We used and validated the Metagenomic and metatranscriptomic techniques used in the present study in a previous report on the salivary microbiota, which was published in 2017 (1).<br><br>1. Belstrom D et. al. Metagenomic and metatranscriptomic analysis of saliva reveals disease-associated microbiota in patients with periodontitis and dental caries. NPJ Biofilms Microbiomes. 2017 Oct 2;3:23. doi: 10.1038/s41522-017-0031-4. eCollection 2017. |
| Randomization | Patients with periodontitis and healthy controls were consecutively enrolled until sufficient sample size was reached. Patients and controls were included based on presence/abscense of periodontitis, which is why randomization could not be used. |
| Blinding | Blinding was not used for the data collection and the analysis |

# Reporting for specific materials, systems and methods

We require information from authors about some types of materials, experimental systems and methods used in many studies. Here, indicate whether each material, system or method listed is relevant to your study. If you are not sure if a list item applies to your research, read the appropriate section before selecting a response.

## Materials & experimental systems

| n/a | Involved in the study | |
|---|---|---|
| ☒ | ☐ | Antibodies |
| ☒ | ☐ | Eukaryotic cell lines |
| ☒ | ☐ | Palaeontology and archaeology |
| ☒ | ☐ | Animals and other organisms |
| ☐ | ☒ | Human research participants |
| ☒ | ☐ | Clinical data |
| ☒ | ☐ | Dual use research of concern |

## Methods

| n/a | Involved in the study | |
|---|---|---|
| ☒ | ☐ | ChIP-seq |
| ☒ | ☐ | Flow cytometry |
| ☒ | ☐ | MRI-based neuroimaging |

## Human research participants

Policy information about studies involving human research participants

| | |
|---|---|
| Population characteristics | The study population included 11 patients with chronic periodontitis, and 11 orally healthy controls, which were enrolled in January 2018 at University of Copenhagen, Department of Odontology.<br>The periodontitis group was comprised of 5 males and 6 females with a mean age of 64 yrs. (55-77 yrs.), whereas the control group included 4 males and 7 females with at mean age of 60 yrs. (50-71 yrs.). |
| Recruitment | Patients with periodontitis and healthy controls were consecutively enrolled until sufficient sample size was reached. |
| Ethics oversight | All 22 participants signed informed consent. The study was approved by the regional ethical committee (H-16016368) and reported to the local data authorization of University of Copenhagen (SUND-2018-8). |

Note that full information on the approval of the study protocol must also be provided in the manuscript.
